# Removal of the C-Terminal Domains of ADAMTS13 by Activated Coagulation Factor XI induces Platelet Adhesion on Endothelial Cells under Flow Conditions

**DOI:** 10.3389/fmed.2017.00232

**Published:** 2017-12-20

**Authors:** Kathleen S. Garland, Stéphanie E. Reitsma, Toshiaki Shirai, Jevgenia Zilberman-Rudenko, Erik I. Tucker, David Gailani, András Gruber, Owen J. T. McCarty, Cristina Puy

**Affiliations:** ^1^Division of Pediatric Hematology/Oncology, School of Medicine, Oregon Health & Science University, Portland, OR, United States; ^2^Department of Biomedical Engineering, School of Medicine, Oregon Health & Science University, Portland, OR, United States; ^3^Department of Clinical Chemistry and Haematology, University Medical Center Utrecht, Utrecht, Netherlands; ^4^Aronora, Inc., Portland, OR, United States; ^5^Vanderbilt University School of Medicine, Nashville, TN, United States

**Keywords:** a disintegrin and metalloproteinase with a thrombospondin type 1 motif, member 13, factor XI, coagulation, von Willebrand factor, platelets

## Abstract

Platelet recruitment to sites of vascular injury is mediated by von Willebrand factor (VWF). The shear-induced unraveling of ultra-large VWF multimers causes the formation of a “stringlike” conformation, which rapidly recruits platelets from the bloodstream. A disintegrin and metalloproteinase with a thrombospondin type 1 motif, member 13 (ADAMTS13) regulates this process by cleaving VWF to prevent aberrant platelet adhesion; it is unclear whether the activity of ADAMTS13 itself is regulated. The serine proteases α-thrombin and plasmin have been shown to cleave ADAMTS13. Based on sequence homology, we hypothesized that activated coagulation factor XI (FXIa) would likewise cleave ADAMTS13. Our results show that FXIa cleaves ADAMTS13 at the C-terminal domains, generating a truncated ADAMTS13 with a deletion of part of the thrombospondin type-1 domain and the CUB1-2 domains, while α-thrombin cleaves ADAMTS13 near the CUB1-2 domains and plasmin cleaves ADAMTS13 at the metalloprotease domain and at the C-terminal domain. Using a cell surface immunoassay, we observed that FXIa induced the deletion of the CUB1-2 domains from ADAMTS13 on the surface of endothelial cells. Removal of the C-terminal domain of ADAMTS13 by FXIa or α-thrombin caused an increase in ADAMTS13 activity as measured by a fluorogenic substrate (FRETS) and blocked the ability of ADAMTS13 to cleave VWF on the endothelial cell surface, resulting in persistence of VWF strands and causing an increase in platelet adhesion under flow conditions. We have demonstrated a novel mechanism for coagulation proteinases including FXIa in regulating ADAMTS13 activity and function. This may represent an additional hemostatic function by which FXIa promotes local platelet deposition at sites of vessel injury.

## Introduction

After vascular injury, the large glycoprotein (GP) von Willebrand factor (VWF) binds to exposed collagen through its A3 domain. VWF multimers circulate in plasma in a globular form under normal flowing conditions. However, VWF unravels into a “string-like” conformation when it is exposed to increased shear forces (1). This reveals the VWF-binding site located within the A1 domain for the platelet receptor GPIbα, resulting in the rapid recruitment and adhesion of platelets to VWF (2). Endothelial cells constitutively secrete VWF as multimers of varying size into the blood. VWF is also stored in endothelial cells within Weibel–Palade bodies, predominantly as “ultra-large” multimers (UL-VWF) (3). The presence of increased levels of UL-VWF multimers in plasma has been shown to initiate the formation of VWF-platelet microthrombi, resulting in debilitating thrombotic complications such as thrombotic thrombocytopenic purpura (TTP) (4). The cause of increased UL-VWF levels in the majority of patients with TTP has been attributed to either congenital defects in a disintegrin and metalloproteinase with a thrombospondin type 1 motif, member 13 (ADAMTS13) or due to the presence of autoantibody inhibitors that compromise the function of ADAMTS13 (4).

ADAMTS13 has a molecular weight of 200 kDa, consisting of a metalloprotease (MET) domain, a disintegrin-like domain, a first thrombospondin type-1 repeat (TSP1) domain, a Cys-rich domain, and a spacer domain (5). Moreover, the C-terminal domain of ADAMTS13 contains an additional seven TSP1 repeats and two CUB domains (6). The hemostatic potential of VWF is regulated through a multistep mechanism of proteolysis of its A2 domain by ADAMTS13. VWF is resistant to cleavage by ADAMTS13 until subjected to fluid shear stress (7), adsorbed on a surface (6, 7), or treated with reagents that cause denaturation (6, 8), all of which “unravel” VWF and permit ADAMTS13 access to the scissile bond within the A2 domain of VWF. ADAMTS13 has been shown to adopt a natural folded conformation, allowing the CUB-1 domains to interact with its spacer domain (9). This generally closed conformation prohibits the functional exosite on the spacer domain from interacting with the proteolytic site on the A2 domain of VWF (9, 10). The binding of the C-terminal TS8-CUB2 domain of ADAMTS13 with the C-terminal D4-CK domain of VWF mediates a conformational activation of ADAMTS13, leading to the exposure of the spacer, cysteine-rich, disintegrin-like, and MET domains exosite, and ultimately increased proteolysis of VWF by ADAMTS13 (11–13). Deletion of the C-terminal TSP1 and CUB domains of ADAMTS13 impairs the cleavage of large VWF multimers *in vitro* (11, 14), increases vascular thrombosis *in vivo* (15), and yet increases the cleavage of peptide substrates such as FRETS-VWF73 (10). These findings suggest that the CUB domains regulate ADAMTS13 activity.

ADAMTS13 is constitutively active and has no known inhibitors *in vivo*. To date, it is still uncertain how ADAMTS13 activity is regulated, and what impact this has on the inactivation of VWF. The serine proteases α-thrombin, activated FX (FXa), and plasmin have been shown to cleave the C-terminal of ADAMTS13 *in vitro* under static conditions (16). Yet, it is still unknown whether the deletion of the C-terminal of ADAMTS13 by α-thrombin or plasmin inhibits the functional activity of ADAMTS13 on the processing of endothelial VWF to affect platelet recruitment and aggregation under flow conditions.

FXI is a contact pathway serine protease that has been shown to play an increasingly relevant role in hemostasis (17). Congenital factor XI deficiencies were reported to exhibit protection from ischemic stroke and to exhibit a lower incidence of venous thromboembolism (VTE) (18, 19), while elevated levels of FXI are an independent risk factor for VTE and ischemic stroke (20, 21). The primary substrate of the serine protease activated FXI (FXIa) in the classic coagulation model is FIX; however, increasing evidence has shown that FXIa promotes thrombin generation by enzymatic activation of FXI, FX, FVIII, FV (22–24), and inactivation of tissue factor pathway inhibitor (TFPI), *in vitro* (25).

Based on sequence homology, we hypothesized that FXIa cleaves and inactivates ADAMTS13 leading to VWF string formation, platelet aggregation, and thrombus formation. In the present study, we developed an endothelialized flow chamber that allowed us to study whether the components of the coagulation cascade can regulate ADAMTS13 activity under flow conditions.

## Materials and Methods

### Reagents

Recombinant ADAMTS13 (rADAMTS13) was donated from Shire (Benatzkygasse, Austria). Plasma-derived FXIa, α-thrombin, plasmin, and FXa were purchased from Haematologic Technologies (Essex Junction, VT, USA). α-FXIIa and kallikrein were from Enzyme Research Laboratories, Inc. (South, IN, USA). Rabbit polyclonal anti-ADAMTS13 antibody, specific for the MET domain, was from Abcam (Cambridge, MA, USA). Rabbit polyclonal anti-ADAMTS13 antibody, specific for the CUB1-2 domains, was from Santa Cruz Biotechnology (Dallas, TX, USA). Goat polyclonal anti-ADAMTS13 antibody, specific for the TSP4 domains, was from Bethyl Laboratories (Montgomery, TX, USA). Hirudin, *O*-phenylenediamine (OPD) substrate, and aprotinin were from Sigma-Aldrich (St. Louis, MO, USA). FRETS-VWF73 (VWF residues 1,596–1,668) was purchased from PeptaNova (Louisville, KY, USA).

### Coomassie Blue Staining and Western Blot

rADAMSTS13 (250 nM) was incubated with FXIa (50 nM) with or without aprotinin (50 µM), and with plasmin, α-thrombin, FXa, FXIIa, or kallikrein at 37°C over a time interval of 0–4 h in 25 mM Hepes, 150 mM NaCl (HBS) pH 7.4. The addition of Ca^2+^ (5 mM) was necessary for proteolytic activity of α-thrombin, plasmin, and FXa. Samples were separated by SDS-PAGE under reducing conditions and analyzed by Coomassie blue staining, or they were transferred to PVDF membrane and immunoblotted with an anti-MET domain antibody, anti-ADAMTS13 CUB1-2 domain antibody, or an anti-TSP4 domain antibody, followed by HRP-conjugated secondary antibodies. Proteins were detected using ECL (GE Healthcare, Piscataway, NJ, USA).

### Cell Surface Immunoassays

Human umbilical vein endothelial cells (HUVECs, ATCC, Manassas, VA, USA) were grown to confluence in a 96-well plate using an endothelial cell basal medium-2 enriched with supplements (Lonza, Walkersville, MD, USA) and incubated with rADAMTS13 (50 nM) for 1 h at 37°C in serum-free medium (SFM, Thermo Fisher Scientific) with BSA (0.1%) and ZnCl_2_ (10 µM). HUVECs were then washed and incubated with FXIa (30 nM), α-thrombin (30 nM), or plasmin (30 nM) for 0, 1, or 2 h at 37°C in SFM with BSA (0.1%) and ZnCl_2_ (10 µM), followed by incubation with 50 µM aprotinin and 10 µg/mL hirudin for 10 min at 37°C. HUVECs were then washed with HBS, fixed with paraformaldehyde (2%), blocked in PBS with tween (0.05%) and BSA (3%), and probed with either the anti-ADAMTS13 CUB antibody (5 µg/mL) or an anti-ADAMTS13 TSP4 antibody (1 µg/mL) for 1 h. This was followed by incubation with an HRP-coupled secondary anti-rabbit antibody (1:2,000) or anti-goat antibody (1:2,000), and then measured with an OPD substrate at an absorbance of 450 nm.

### ADAMTS13 Activity Assay

rADAMSTS13 (30 nM) was incubated with either FXIa (5 nM), plasmin (5 nM), α-thrombin (5 nM), or vehicle for 3 h at 37°C in HBS. Aprotinin (50 µM) and hirudin (10 µg/mL) was added to each sample and allowed to sit at RT for 10 min. rADAMTS13 was diluted to 4 nM in reaction buffer (5 mM Bis-Tris, 25 mM CaCl_2_, 0.005% Tween; pH 6.0). The reaction was initiated by the addition of an equal volume of 4 µM FRETS-VWF73 substrate. Fluorescence (excitation, 340 nm; emission, 460 nm) was measured at 1-min intervals for 1 h using a fluorescence spectrometer.

### Flow Experiment and Staining

HUVECs were cultured in the parallel-plate flow chamber (ibidi μ-slide VI^0.1^) under shear conditions beginning 48 h before the flow experiment. Devices were incubated with cells for 30 min prior to induction of passive flow by adding growth medium to the inflow port of each channel. Passive flow was maintained by emptying the outflow and refilling the inflow for 1 h or until a morphology changes in HUVECs from round to elongated was noted. Inflow ports of the flow devices were then connected to a growth medium well and the outflow ports were connected to a waste well. All six channels were connected to the same growth medium well and the outflowing growth medium was recirculated. The height of the medium well was maintained to achieve an initial shear stress of 1.54 dyne/cm^2^ for 48 h or until confluence.

Cells were starved in SFM for 2 h, and then stimulated with 10 ng/mL TNFα in SFM supplemented with 3% fatty acid-free BSA for 4 h. Human whole blood was drawn into 3.8% sodium citrate from healthy donors per institutional IRB protocol and washed platelets were purified as previously described (26). Washed platelets (3 × 10^8^/mL) in the presence or absence of rADAMTS13 (2.5 nM) were perfused through the flow chamber for 10 min at 2.5 dyne/cm^2^. Samples were fixed with 4% PFA overnight at 4°C. Platelet-VWF string formation was visualized by labeling with mouse anti-human CD41-FITC (ThermoFisher Scientific, Grand Island, NY, USA) and rabbit anti-human VWF antibody, respectively, and AlexaFluor-labeled secondary antibodies (Thermo Fisher Scientific, Grand Island, NY, USA). Five random fields at 20× magnification were imaged for each chamber by using a Zeiss Axiovert 200M microscope and SlideBook 6 software. The number of platelet-VWF string formations were manually counted. UL-VWF length was analyzed by using ImageJ software. Statistical significance was evaluated using the Dunnett’s multiple comparison test, and *p*-values <0.05 were considered to be statistically significant.

## Results

### Proteolytic Cleavage of ADAMTS13 by FXIa

**Figure 1:**
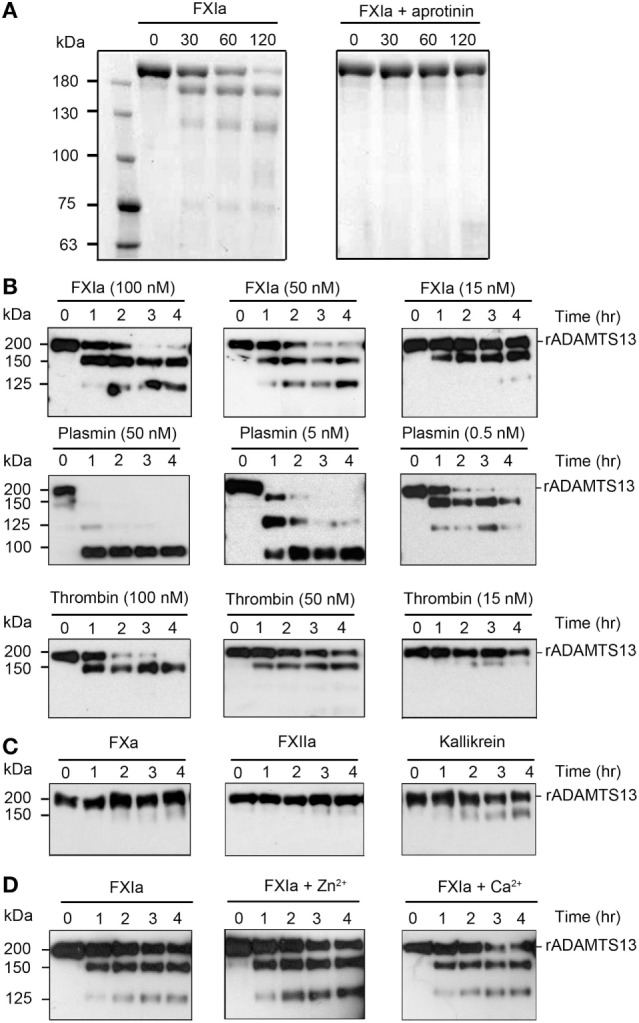
Proteolysis of ADAMTS13 by FXIa. **(A)** rADAMTS13 (250 nM) was incubated with FXIa (50 nM), in the absence or presence of aprotinin (50 µM) for selected times (0–120 min) at 37°C before being separated by SDS-PAGE under reduced conditions and analyzed by Coomassie blue staining. rADAMTS13 fragment size (kDa) is shown following proteolysis by FXIa **(B)** rADAMTS13 (250 nM) was incubated with FXIa (100–15 nM), plasmin (50–0.5 nM), or α-thrombin (100–15 nM) for selected times (0–4 h) at 37°C. rADAMTS13 was analyzed by western blotting using an anti-ADAMTS13 MET domain antibody **(C)** rADAMTS13 (250 nM) was incubated with FXa (50 nM), FXIIa (50 nM), and kallikrein (50 nM) for selected times (0–4 h) at 37°C. rADAMTS13 was analyzed by western blotting using an anti-ADAMTS13 MET domain antibody **(D)** rADAMTS13 (250 nM) was incubated with FXIa (50 nM) for selected times (0–4 h) at 37°C before being analyzed by western blotting using an anti-ADAMTS13 MET domain antibody in the absence or presence of CaCl_2_ or ZnCl_2_ (*n* = 3).

Previous studies have shown proteolytic cleavage and inactivation of ADAMTS13 by the known serine proteases plasmin and α-thrombin (16). Herein, we investigated whether FXIa was able to proteolyze ADAMTS13. We observed *via* SDS-PAGE analysis that incubation of rADAMSTS13 with FXIa for 2 h led to the disappearance of the ~200 kDa ADAMTS13 band and the appearance of lower molecular weight bands under reducing conditions (Figure 1A). The presence of aprotinin, which inhibits FXIa activity, blocked the degradation of ADAMTS13 by FXIa (Figure 1A). Then, we compared the capacity of FXIa to proteolyze ADAMTS13 relative to plasmin and α-thrombin. In accordance with previous studies (16), we observed that plasmin rapidly degraded ADAMTS13; in contrast, 15 nM α-thrombin barely cleaved ADAMTS13 after 4 h of incubation (Figure 1B). FXa, Kallikrein, or FXIIa were not able to cleave ADAMTS13 under the conditions tested here (Figure 1C). The addition of Ca^2+^ was necessary for proteolytic activity of α-thrombin and plasmin; in contrast, neither Ca^2+^ nor Zn^2+^ were required for the proteolytic cleavage of ADAMTS13 by FXIa (Figure 1D).

### FXIa Deletes the TSP6-8 Domains and the C-Terminal CUB1-2 Domains of ADAMTS13

**Figure 2:**
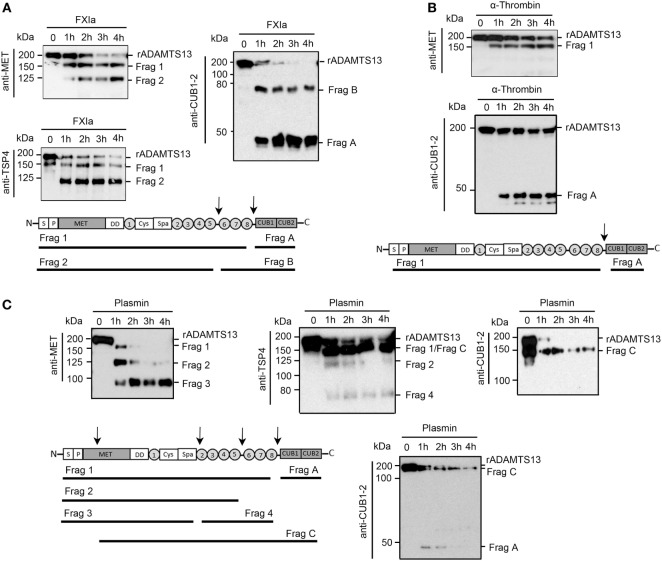
Characterization of ADAMTS13 proteolysis by FXIa, α-thrombin, and plasmin. rADAMTS13 (250 nM) was incubated with **(A)** FXIa (50 nM), **(B)** α-thrombin (50 nM), or **(C)** plasmin (5 nM) for selected times (0–4 h) at 37°C before being analyzed with three different antibodies: anti-ADAMTS13 MET domain antibody, anti-ADAMTS13 CUB1-2 domain antibody, and anti-TSP4 domain antibody. The predicted identities of the formed fragments upon cleavage of rADAMTS13 by FXIa in **(A)**, or by α-thrombin in **(B)** or by plasmin in **(C)** are depicted in a schematic overview (*n* = 3).

Experiments were next designed to identify the fragments of rADAMTS13 generated by FXIa, α-thrombin, and plasmin. rADAMTS13 (250 nM) was incubated with FXIa (50 nM), α-thrombin (50 nM), or plasmin (5 nM) for increasing times (0–4 h) at 37°C before being analyzed by western blot using an anti-ADAMTS13 antibody against the MET domain (N-terminal), the TSP4 domains, or the two CUB domains (C-terminal). We observed that FXIa caused the disappearance of the ~200 kDa ADAMTS13 band and the appearance of first a band at ~150 kDa (Fragment 1) and then a second band at ~125 kDa (Fragment 2) when the samples were analyzed with the anti-MET domain antibody or an anti-TSP4 domain antibody (Figure 2A). A band at ~50 kDa (Fragment B) and a fragment at ~75 kDa (Fragment A) appeared when the samples were analyzed with an anti-CUB1-2 domain antibody (Figure 2A). These data suggest that FXIa cleaves ADAMTS13 at two sites, first near the two CUB domains, generating a band ~150 kDa and a band ~50 kDa, and then near the TSP6-8 domain, generating a band ~125 kDa (Figure 2A). The location of these fragments is predicted in Figure 2A.

We observed that α-thrombin was only able to cleave ADAMTS13 near the CUB1-2 domain after 4 h of incubation, generating one band at ~150 kDa when the sample was analyzed with the anti-MET domain antibody (Figure 2B), suggesting that α-thrombin can cleave ADAMTS13 at only one site near the two CUB domains. In contrast to FXIa, plasmin was able to generate a third band at ~100 kDa (Fragment 3) when the samples were analyzed with the anti-MET domain antibody. This band around 100 kDa was not detected when the samples were analyzed with the anti-TSP4 domain antibody; instead, an extra band at ~70 kDa (Fragment 4) was detected (Figure 2C). These results indicate that plasmin cleaves ADAMTS13 at multiple sites, not only at the C-terminal domain but also after the spacer domain. When the samples were analyzed with an anti-CUB1-2 domain antibody, plasmin generated a band at ~50 kDa at 1 h of incubation, which disappeared after 4 h of incubation, suggesting that plasmin cleaves at the CUB domain, destroying the binding of the anti-CUB domain antibody. Also, the anti-CUB domain antibody detected a band at 150 kDa, indicating that plasmin may cleave ADAMTS13 at the MET domain located near the N-terminal domain of ADAMTS13 (Figure 2C). The location of these fragments is predicted in Figure 2C.

### FXIa Cleaves the CUB1-2 Domain of ADAMTS13 on the Endothelial Surface

**Figure 3:**
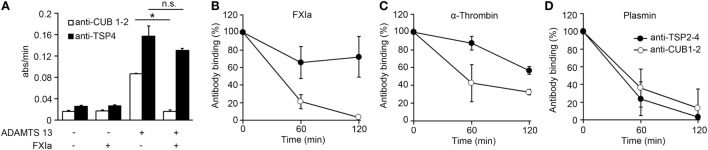
FXIa cleaves the CUB1-2 domain of ADAMTS13 on the endothelial surface. **(A)** HUVECs were incubated with or without rADAMTS13 (50 nM) for 1 h at 37°C, washed, and treated with or without FXIa (30 nM) for 2 h. Reactions were stopped with aprotinin (50 µM), followed by cell surface detection of ADAMTS13 by using either an anti-ADAMTS13 CUB1-2 domain antibody or an anti-ADAMTS13 TSP4 domain antibody. **(B)** HUVECs incubated with rADAMTS13 (50 nM) treated with FXIa (30 nM), **(C)** α-thrombin (30 nM), or **(D)** plasmin (30 nM) for 0–2 h at 37°C. Reactions were stopped with aprotinin (50 µM) and hirudin (10 µg/mL), followed by cell surface detection of ADAMTS13 by using either an anti-ADAMTS13 CUB1-2 domain antibody (○) or an anti-ADAMTS13 TSP4 domain antibody (●). Data are mean ± SE (*n* = 3).

Prior studies have shown that ADAMTS13 binds endothelial cells in a specific manner and that the cleavage of VWF by ADAMTS13 occurs mainly on the EC surface (27). In order to determine if the deletion of the ADAMTS13 C-terminal domain by FXIa or α-thrombin could take place on the surface of ECs, we performed a cell surface immunoassay using an anti-CUB1-2 domain antibody and an anti-TSP4 domain antibody. We incubated HUVECs with rADAMTS13 and measured the binding of both antibodies following incubation of the cells with the serine protease FXIa. We observed that both antibodies only bound to the endothelial cell surface in the presence of rADAMTS13, indicating that both antibodies are specific for ADAMTS13 (Figure 3A). The detection of ADAMTS13 CUB1-2 domains on HUVECs was lost over a period of 2 h following incubation with FXIa (Figures 3A,B). In contrast, FXIa treatment induced a slight decrease in binding of the anti-TSP4 domain antibody (Figures 3A,B). Incubation with α-thrombin only slightly decreased the binding of either the anti-CUB1-2 domain antibody or the anti-TSP4 domain antibody (Figure 3C). In contrast, incubation with plasmin dramatically decreased the binding of antibodies to the ADAMTS13 CUB1-2 domain or the ADAMTS13 TSP4 domain (Figure 3D). These results suggest that FXIa is able to remove the CUB1-2 domains of ADAMTS13 on the surface of ECs. α-thrombin weakly cleaved ADAMTS13 on the endothelial surface, whereas plasmin was able to cleave ADAMTS13 at multiple sites, including the N-terminal domain of ADAMTS13.

### Deletion of the TSP6-8 and the C-Terminal CUB1-2 Domains of ADAMTS13 by FXIa Induces an Allosteric Conformation of ADAMTS13

**Figure 4:**
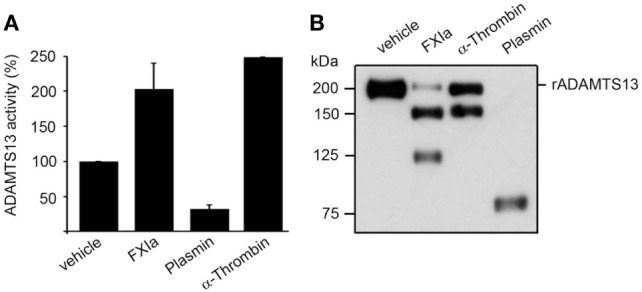
ADAMTS13 activity following proteolysis by FXIa. **(A)** rADAMTS13 (30 nM) was incubated at 37°C for 4 h with the following: FXIa (5 nM), plasmin (5 nM), or α-thrombin (5 nM) in HBS with 5 mM CaCl_2_. Reactions were stopped with aprotinin (50 µM) and hirudin (10 µg/mL). rADAMTS13 was diluted to 4 nM in reaction buffer (5 mM Bis-Tris pH 6.0, 25 mM CaCl2, and 0.005% Tween-20) and the reaction was initiated by the addition of an equal volume of FRETS-VWF73 substrate (4 µM). Data are mean ± SE (*n* = 3). **(B)** Western blot of the samples using an anti-ADAMTS13 MET domain antibody to confirm proteolytic cleavage of ADAMTS13 by the proteases.

It has been shown that truncation of ADAMTS13 after the TSP2-8 domain or antibodies against the CUB1-2 domains induces a conformational change of ADAMTS13, causing ADAMTS13 to unfold fully and expose the spacer domain, resulting in enhanced cleavage of the peptide substrate FRETS-VWF73 (10). Based on the fact that both FXIa and α-thrombin cleave ADAMTS13 near the CUB1-2 domains as described above, experiments were designed to determine whether FXIa or α-thrombin induced an allosteric conformation of ADAMTS13. We compared this with the serine proteases plasmin, which can also cleave ADAMTS13 at the MET domain (16). We observed that after incubation of rADAMTS13 with FXIa at 37°C for 3 h, the activity of ADAMTS13 was increased as measured by the FRETS-VWF73 assay (Figure 4). Analysis of the samples by western blot using an anti-ADAMTS13 MET domain antibody confirmed the generation of 150 and 125 kDa fragments following incubation with FXIa. α-thrombin also was able to increase ADAMTS13 activity toward the peptide substrate FRETS-VWF73. In contrast, plasmin reduced ADAMTS13 activity toward the peptide substrate FRETS-VWF73 due to its capacity to cleave ADAMTS13 at multiple places. These data suggest preferential proteolytic cleavage near the CUB domains of ADAMTS13 by FXIa or α-thrombin, allowing ADATMS13 to adopt a more open configuration and exposing the proteolytic site on ADAMTS13 responsible for cleaving FRETS-VWF73.

### Deletion of the TSP6-8 and the Two C-Terminal CUB Domains of ADAMTS13 by FXIa Inhibits ADAMTS13 Activity Resulting in Increased Platelet Adhesion under Flow

**Figure 5:**
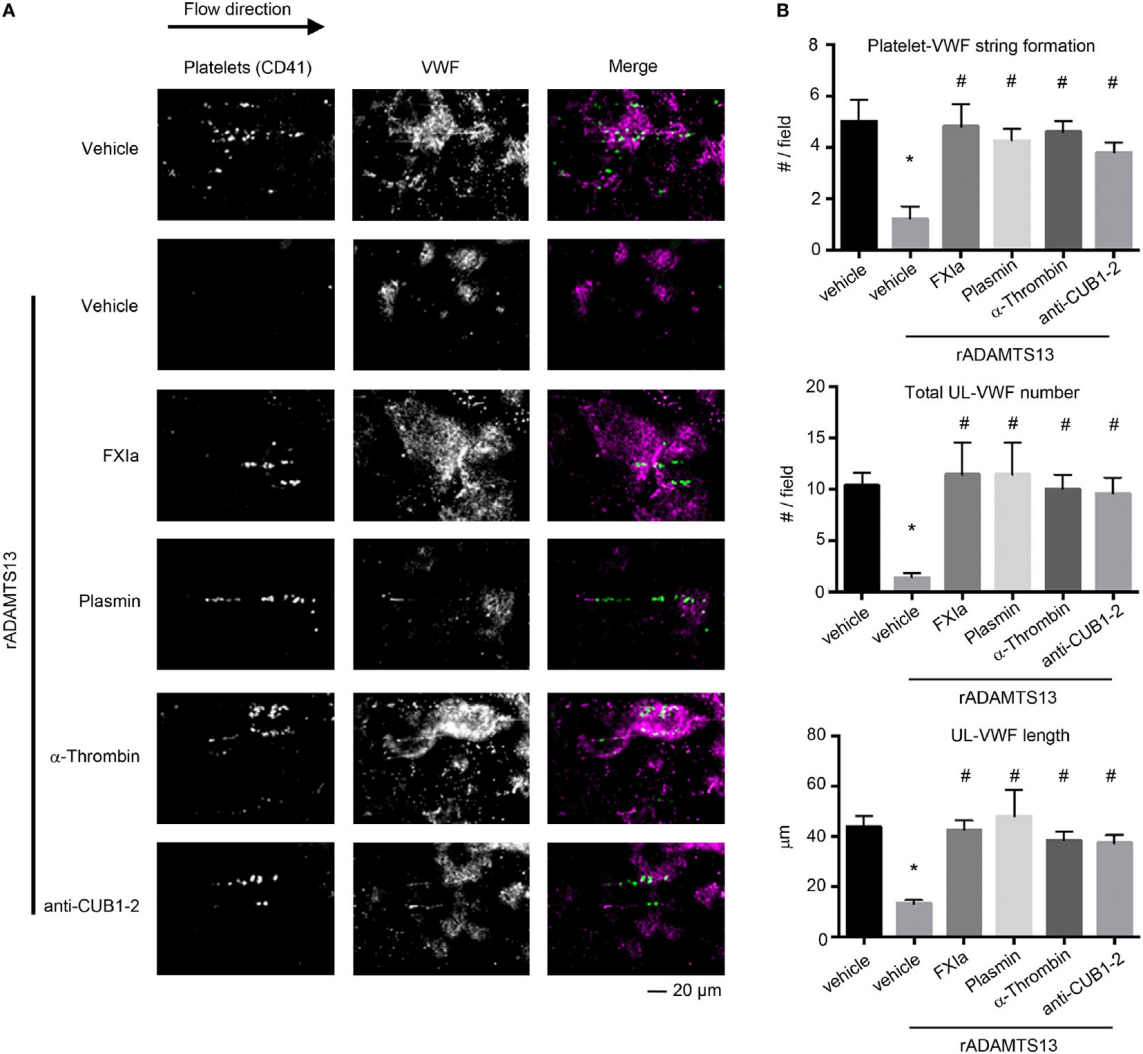
FXIa inhibits ADAMTS13 cleavage of VWF. rADAMTS13 (250 nM) was incubated at 37°C for 4 h with the following: FXIa (50 nM), α-thrombin (50 nM), and plasmin (50 nM) in HBS with 5 mM CaCl_2_. Reactions were stopped with aprotinin (50 µM) and hirudin (10 µg/mL). Endothelialized parallel-plate flow chambers were prepared and EC’s were stimulated with TNFα to release VWF as described above. **(A)** VWF string formation and platelet adhesion depicted with fluorescence following perfusion of washed platelets at a venous flow rate of 2.5 dyne/cm^2^ in the absence or presence of rADAMTS13 (2.5 nM) incubated with either vehicle, FXIa, α-thrombin, plasmin, or an anti-ADAMTS13 CUB domain antibody (20 ng/mL). **(B)** Quantification of platelet string formation, total VWF number, and VWF length compared between noted substrates. Using Dunnett’s multiple comparison test, * and ^#^ indicate statistical significance (*p* < 0.05). Data are mean ± SE (*n* = 3).

It has been proposed that the binding of the ADAMTS13 TSP7-CUB2 domain to the VWF D4CK domains induces a conformational activation of ADAMTS13, causing ADAMTS13 to unfold fully and expose the spacer domain (28). The spacer domain can then directly interact with the VWF A2 domain, enhancing the cleavage of VWF by ADAMTS13 under flow conditions. Besides the fact that removal of the C-terminal of ADAMTS13 domains or antibodies against the CUB1-2 domains enhance the cleavage of the peptide substrate FRETS-VWF73 (10), removal of the C-terminal of ADAMTS13 abrogate the capacity of ADAMTS13 to bind and cleave VWF under flow conditions *ex vivo* (12) and *in vivo* (15). Thus we next determined whether cleavage of the TSP6-8 and the two C-terminal CUB domains of ADAMTS13 by FXIa or the cleavage of the CUB 1-2 domains of ADAMTS13 alone by α-thrombin might abrogate ADAMTS13 activity under flow by assessing the length of VWF that had been released by activated endothelial cells under shear. HUVECs were preincubated with TNFα to induce VWF release and the formation of platelet-VWF strings, the number and the length of UL-VWF were then quantified. We observed that the addition of 2.5 nM full-length rADAMTS13 abrogated platelet-VWF string formation (Figures 5A,B). In contrast, the incubation of rADAMTS13 with FXIa reversed the ability of ADAMTS13 to cleave VWF, resulting in an increase in the formation of platelet-VWF strings. A similar effect was observed when rADAMTS13 was incubated with either α-thrombin or plasmin. Interestingly, the addition of an anti-ADAMTS13 CUB1-2 domain antibody also blocked ADAMTS13 activity under flow (Figures 5A,B).

Utilizing this *in vitro* endothelialized flow chamber technique, our data suggest a novel mechanism by which the activity of ADAMTS13 is regulated: proteolysis by the serine proteases FXIa, α-thrombin, and plasmin inhibits the ability of ADAMTS13 to cleave VWF, promoting platelet-VWF string formation on inflamed endothelial cells under shear flow.

## Discussion

In this study, we have shown a novel mechanistic role by which the serine protease FXIa may regulate platelet deposition at sites of endothelial cell damage by inactivating ADAMTS13. This finding further expands the classical pathway by which activation of the contact pathway of coagulation promotes thrombus formation. FXIa is known to promote thrombin generation through direct activation of FIX, FX, FV, and FVIII (22–24) and inactivation of TFPI, *in vitro* (25). These alternative pathways explain in part why mice lacking both FIX and FXI are more resistant to chemical injury-induced arterial thrombosis than are mice deficient in FIX alone (29). These observations suggest that the role of FXIa in hemostasis and thrombus formation may include activities that bypass the FIX-mediated intrinsic pathway of thrombin generation. Here, we show not only that FXIa cleaves and inactivates ADAMTS13 leading to platelet aggregation along persistent VWF strands under flow but that this interaction can occur on the endothelial surface.

ADAMTS13 has been shown to adopt a closed conformation due to an interaction between its CUB-1 domain and spacer domain (10). Following binding to VWF and under shear flow, ADAMTS13 unfolds and the TS8-CUB2 domain binds to the D4-CK domain on the elongated VWF strand (11, 13). The spacer domain of ADAMTS13 can then bind to the VWF A2 domain leading to the cleavage of VWF. To date, there are no known endogenous inhibitors of ADAMTS13 activity, which would in turn promote persistence of ultra-long VWF strands and resultant platelet aggregation at sites of endothelial injury. Prior studies have demonstrated that the serine proteases α-thrombin and plasmin are able to cleave ADAMTS13, abolishing its enzymatic activity toward purified human VWF *in vitro* (16). Here, we observed that plasmin cleaves ADAMTS13 at multiple sites and more rapidly than α-thrombin, confirming findings from previous reports (16). Previous work has shown that 9 nM α-thrombin is capable of rapid proteolysis of full-length ADAMTS13 (16). Under the conditions used in our study, we found that the proteolysis of ADAMTS13 by α-thrombin was slow at higher concentrations, and almost non-existent at 15 nM. While we observed that α-thrombin preferentially cleaves ADAMTS13 just before the CUB1-2 domains, FXIa appears to cleave ADAMTS13 at the C-terminal domain at two sites. The first cleavage site appears to be located near to the start of the first CUB domain, producing a fragment of ~150 kDa, while the second cleavage site occurs near to the start the TSP6 domain, producing a fragment of ~125 kDa. This suggests that FXIa removes the TSP6-8-CUB1-2 domain of ADAMTS13.

Plasmin shows a preference for lysine at the P1-position for substrate cleavage; in contrast, thrombin and FXIa have a P1 preference for arginine. While FXa also has a P1 preference for arginine, it shows preference for glycine at the P2-position. However, thrombin has a strict preference for proline at the P2-position, while FXIa has preference for either proline or threonine at the P2-position (30). This could explain the difference in the ADAMTS13 cleavage site selectively between the serine proteases. Also, unlike plasmin or α-thrombin, the cleavage of ADAMTS13 by FXIa does not require the presence of Ca^2+^. In contrast, activation of FIX by FXIa is a calcium-dependent process. The FIX gamma-carboxyglutamic (Gla) domain requires the binding of calcium ions in order to bind to the apple 3 domain of FXIa (31). Previously, we demonstrated that FXIa also can activate FX and FV and cleave TFPI, albeit that these reactions are less efficient as compared to the activation of FIX by FXIa. However, Ca^2+^ was not a requirement for any of these reactions (22, 23, 25). These results suggest that only FIX requires the binding of Ca^2+^ to the Gla domain for the interaction with FXIa, while the other FXIa substrates do not need the binding of calcium ions to interact with FXIa.

UL-VWF multimers released by endothelial cells and elongated under high shear flow conditions typically undergo rapid proteolysis by ADAMTS13. *In vitro* studies have suggested that the endothelial cell surface accelerates the cleavage of VWF strands, as the endothelium serves as an anchor for ADAMTS13 (27). We sought to determine whether FXIa, α-thrombin, or plasmin cleaves ADAMTS13 on the endothelial surface. We showed the C-terminus of ADAMTS13 was removed following proteolysis by all three serine proteases, most notably FXIa. Importantly, this work shows a role for the endothelium in facilitating the inactivation of ADAMTS13 by FXIa.

**Figure 6:**
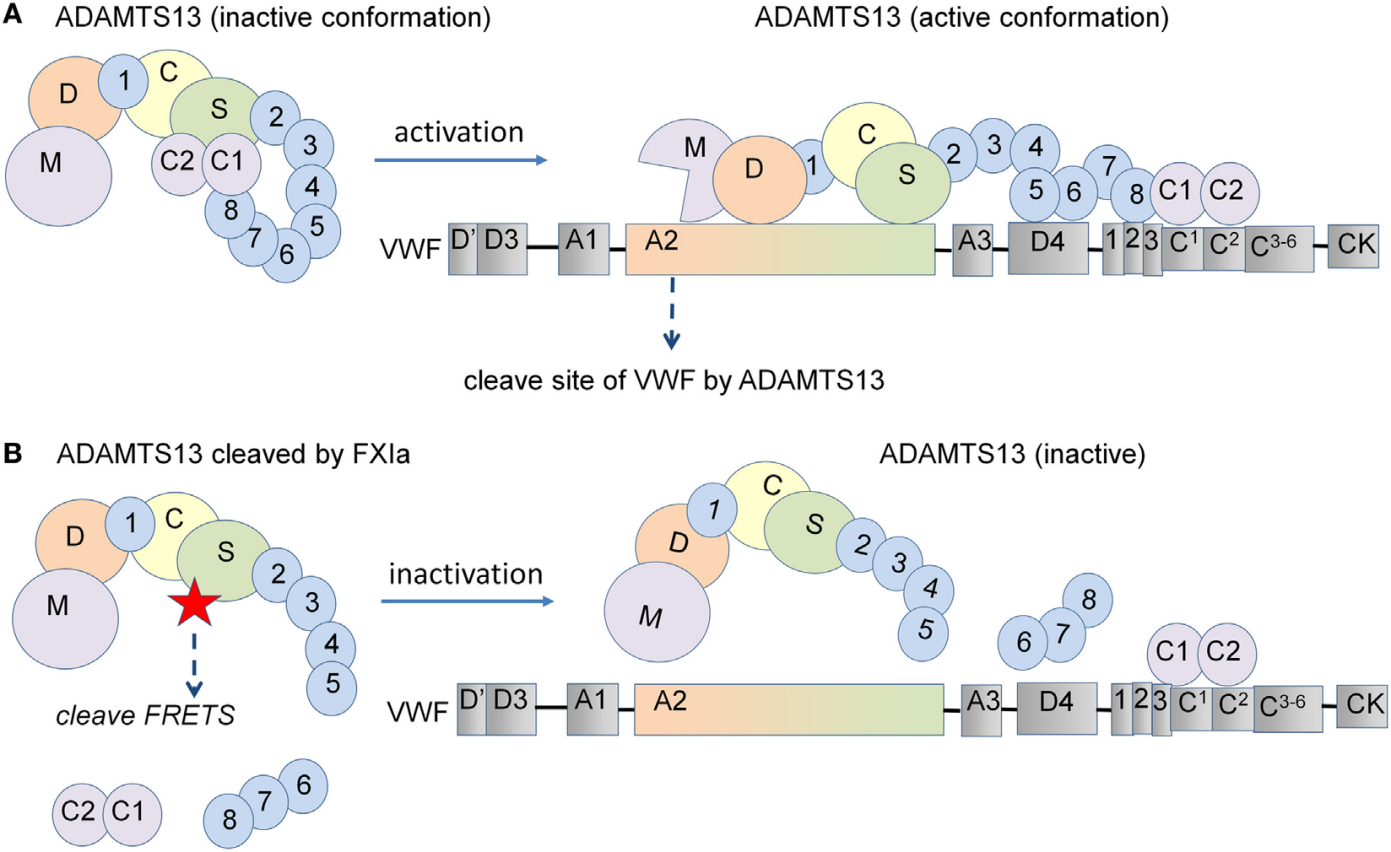
Schematic representation of the proposed model for ADAMTS13 substrate cleavage. **(A)** ADAMTS13 circulates in plasma in a closed conformation due to an interaction between its CUB-1 domain and the spacer domain. Following the binding of the ADAMTS13 TSP7-CUB2 domain to the VWF D4CK domains under shear flow, ADAMTS13 unfolds and the spacer domain of ADAMTS13 becomes available for binding to the A2 domain of VWF, leading to the efficient cleavage of VWF. **(B)** However, deletion of the C-terminal domains of ADAMTS13 induce a conformational change, increasing the cleavage of the FRETS-VWF73 peptide substrate under static conditions. In contrast, the removal of the C-terminal TSP1 and CUB domains from ADAMTS13 decreases the binding of ADAMTS13 to VWF and also decreases its capacity to cleave VWF when subjected to shear flow. In this study, we show that the removal of the C-terminal domains of ADAMTS13 by the serine proteases FXIa and α-thrombin enhance the capacity of ADAMTS13 to cleave the FRETS peptide substrate under static conditions and abolished the ability to cleave VWF strings on endothelial cells under flow conditions.

It has been shown that a rADAMTS13 mutant lacking the C-terminal TSP1 and CUB domains maintains the same VWF-cleaving activity as wild-type rADAMTS13, yet, cleaves VWF even more efficiently under static conditions. This indicates that the C-terminal domains are dispensable for ADAMTS13 activity under these conditions (12). Interestingly, the same study shows that removal of the C-terminal TSP1 and CUB domains results in a marked decrease in VWF-ADAMTS13 binding and cleavage of VWF by ADAMTS13 when subjected to shear flow (12). Synthetic peptides derived from the first CUB domain have been shown to inhibit the cleavage of VWF multimers by ADAMTS13 on endothelial cells under flow (13), while removal of the TSP7-CUB1-2 domains has been shown to accelerate thrombus growth *in vivo* (15). These results indicate that the C-terminal domain of ADAMTS13 plays a crucial role in the recognition and cleavage of VWF. However, deletion of the distal ADAMTS13 domain or use of monoclonal antibodies against the C-terminal domains showed accelerated cleavage of the peptide substrate FRETS-VWF73 due to a conformational change in ADAMTS13 (10). Here, we have shown that the removal of the ADAMTS13-CUB domains by the serine proteases FXIa and α-thrombin enhances the capacity of ADAMTS13 to cleave the peptide substrate FRETS and blocks its ability to cleave VWF strings on endothelial cells (Figure 6). This resulted in increased platelet adhesion to endothelial cells under flow conditions, suggesting that cleavage of ADAMTS13 by either FXIa or α-thrombin likely reduces ADAMTS13 binding to VWF under flow conditions. We show that plasmin cleaves ADAMTS13 at multiple sites, confirming that this induces a decrease in the cleavage of FRETS-VWF73 as previously reported (32) and the cleavage of VWF under flow conditions. Our results suggest that removal of the C-terminal TSP1 and CUB domains by FXIa and α-thrombin may limit ADAMTS13-mediated VWF inactivation *in vivo*.

Circulating plasma proteins play an important role in the delicate balance between hemostasis and thrombosis when damage to the endothelium occurs. Congenital deficiency of FXI, also known as Hemophilia C, typically causes mucosal bleeding symptoms or presents as increased bleeding in patients who have undergone a surgical procedure (17). There is poor correlation between measured FXI plasma levels and bleeding symptoms. Interestingly, studies have observed an association between bleeding tendency in partial FXI deficiency and VWF plasma levels (33, 34). The only known mechanism of action for ADAMTS13 is regulating VWF length. Our results lead us to hypothesize an additional hemostatic mechanism of action for FXIa, with regards to regulation of ADAMTS13 activity. It has been shown that ADAMTS13 and FXI complexes are not found in plasma, suggesting that a circulating complex between these two proteins is unlikely to play a role in normal hemostasis or in the pathophysiology of TTP (35). However, FIX, the main substrate for FXIa does not bind to zymogen FXI, either. FIX binds to an exosite on the apple 3 domain of FXIa, indicating that binding sites are unmasked by a conformational change when FXI is activated (36). Along these lines, we recently found that FXIa is able to cleave TFPI despite the fact that the zymogen FXI is not able to bind to TFPI; rather, only the active form of FXI is able to bind to TFPI (25).

Several studies have shown that elevated levels of FXI promote thrombosis, where patients are at increased risk for VTE and ischemic stroke (20, 21). Experimental thrombosis is reduced in mice lacking the contact pathway factors FXII, HK, PK, or FXI (29). A recent clinical study demonstrated that reducing FXI levels served as an effective anticoagulation regimen for preventing postoperative VTE (37). Thrombus formation can have morbid consequences when ADAMTS13 activity is inhibited by the presence of autoantibodies, as in TTP, or when ADAMTS13 is absent, as in congenital TTP (4). Moreover, reduced activity of ADAMTS13 toward VWF has been suggested to promote both ischemic stroke and myocardial infarction (38, 39). Herein, we show a possible procoagulant mechanism through which FXIa, like the other serine proteases, downregulates ADAMTS13 activity to promote platelet aggregation along persistent VWF strands. Specifically, we show that FXIa-mediated deletion of the ADAMTS13-CUB domains leads to inactivation of ADAMTS13 and resultant persistence of VWF-platelet strings under flow conditions. This interaction suggests a mechanism by which elevated levels of FXI may put patients at a higher risk for VTE events and provides a basis for directing targeted therapeutics in debilitating thrombotic diseases.

## Ethics Statement

This study was carried out in accordance with the recommendations of Oregon Health & Science University Institutional Review Board committee with written informed consent from all subjects. All subjects gave written informed consent in accordance with the Declaration of Helsinki. The protocol was approved by the Oregon Health & Science University Institutional Review Board committee.

## Author Contributions

SR, KG, ET, DG, AG, OM, and CP designed research and wrote the manuscript; KG, TS, JZ-R, and SR performed research; and SR, KG, ET, DG, AG, OM, and CP analyzed and interpreted data.

## Conflict of Interest Statement

AG, ET, and Oregon Health & Science University have a significant financial interest in Aronora Inc., a company that may have a commercial interest in the results of this research. This potential conflict of interest has been reviewed and managed by the Oregon Health & Science University Conflict of Interest in Research Committee. The remaining authors declare no competing financial interests.
